# Intravesical stone formation several years after hysterectomy: a case report

**DOI:** 10.1186/1752-1947-7-230

**Published:** 2013-10-02

**Authors:** Chih-Ming Lu

**Affiliations:** 1 Department of Urology, Buddhist Dalin Tzu Chi Hospital, Chiayi 62247, Taiwan

**Keywords:** Bladder calculi, Lower urinary tract symptoms, Non-absorbable suture

## Abstract

**Introduction:**

Most bladder stones develop in patients with bladder outlet obstruction. Intravesical stone formation after surgery outside the urinary bladder is rare.

**Case presentation:**

A 54-year-old Taiwanese woman with lower urinary tract symptoms following a hysterectomy 14 years ago presented to our hospital. The intravesical calculus had developed from non-absorbable sutures and hung on the dome of the urinary bladder. The stone and residuum of the suture were retrieved by performing an endoscopic procedure.

**Conclusions:**

The presence of an intravesical stone should be suspected in patients with a history of hysterectomy who have symptoms in the lower urinary tract. A hanging stone on the dome of the urinary bladder implies that suture materials migrate into the urinary bladder. The complication can be prevented by the routine use of absorbable material and double-checking with cystoscopy.

## Introduction

Most bladder stones are found among patients who are bedridden, have a urethral catheter, bladder outlet obstruction, infection and other, similar characteristics [1]. On serial kidney-ureter-urinary bladder (KUB) X-ray films, the radiopaque shadow of bladder calculus usually moves while the patient is repositioned. A radiolucent bladder stone can potentially be missed by plain radiography, but can be visualized by ultrasonography. Most immovable vesical stones are found in reconstructive procedures of the urinary bladder [2]. In addition, several investigators have reported that intrauterine contraceptive devices migrate through the wall of the urinary bladder and then serve as a nidus of intravesical stone formation [3-6]. However, immovable bladder stone is quite rare in patients who have never had reconstructive surgery of the urinary bladder. This article reports the case of a woman who developed an intravesical stone 14 years after hysterectomy.

## Case presentation

A 54-year-old Taiwanese woman presented to our emergency department with a long history of dysuria, urinary frequency and pain on voiding. She had a history of hysterectomy for uterine myoma at the age of 40. She sought medical aid for these symptoms at the gynecological department, but without resolution.

**Figure 1:**
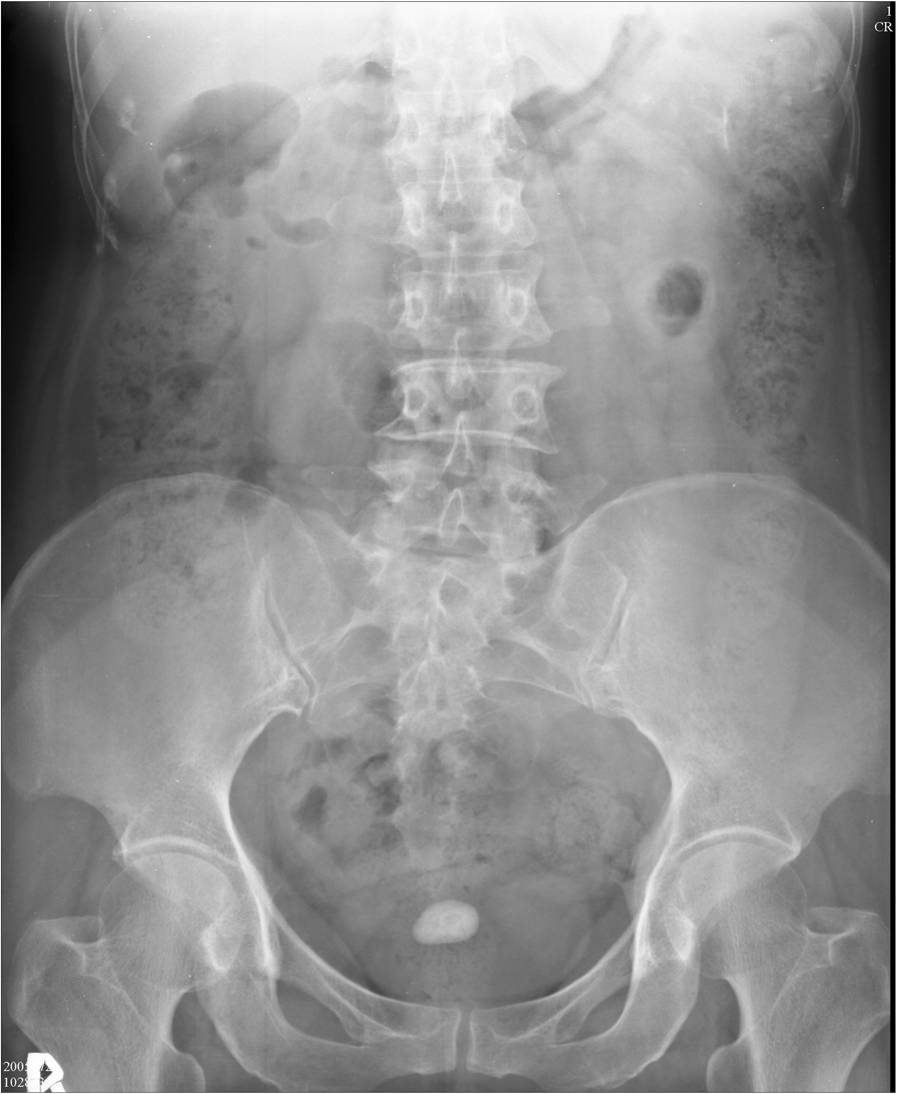
Abdominal X-ray showing a radiopaque shadow of 2.7cm × 1.5cm over the pelvis.

A physical examination revealed mild tenderness over the suprapubic area and no evidence of uterine prolapse. Laboratory test results demonstrated that her serum creatinine level was 11mg/dl; urinalysis showed 6 to 8 red blood cells per high-power field (HPF), 8 to 10 white blood cells per HPF and tiny calcium oxalate crystals; and the blood cell count was within normal range. The KUB film revealed a radiopaque shadow of 2.7cm × 1.5cm size over the pelvis (Figure 1). Cystoscopy showed a hanging stone on the dome of the urinary bladder.

**Figure 2:**
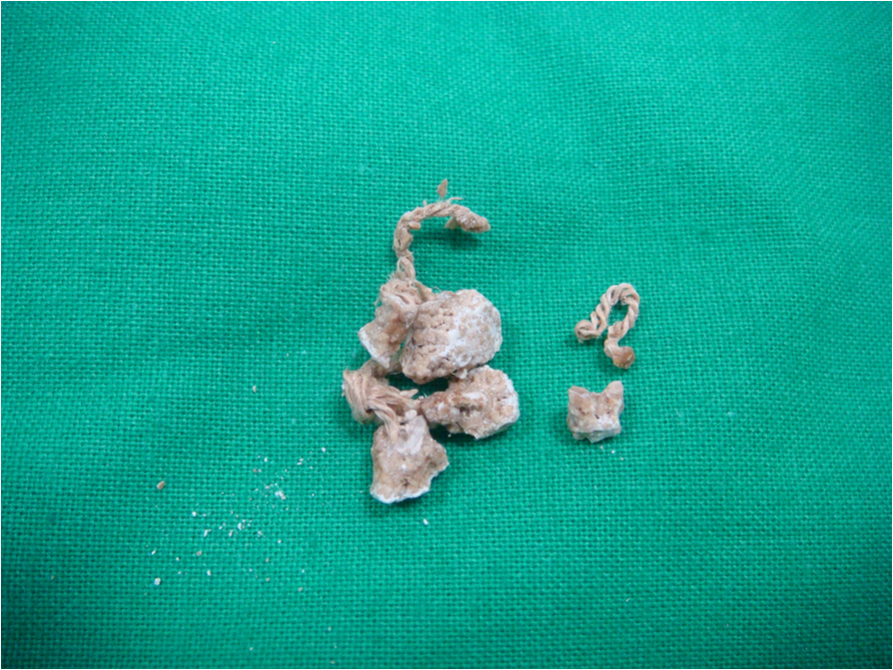
Image showing that the bladder stone encrusted the non-absorbable suture.

The patient later underwent cystolithotripsy with the Swiss LithoClast® lithotripter tool (Boston Scientific, Natick, MA, USA) while under spinal anesthesia. The bladder stone was found to be attached to the bladder wall by a non-absorbable suture (Figure 2). The stone and suture material were retrieved endoscopically. The stone was 0.455mg in weight. The result of stone analysis was calcium oxalate. The suture material was a multi-strand silk thread 3.4cm in length. She was discharged on the next day. Her lower urinary tract symptoms subsided soon afterward. In 5 years of follow-up, there was no postoperative complication.

## Discussion

Several surgeons have reported that suture materials served as a nidus for intravesical stones after patients underwent reconstruction procedures of the lower urinary tract [7-9]. In general, this complication develops after application of non-absorbable sutures using anastomotic techniques. In addition, incidental bladder wall injury during surgical procedures may promote further bladder wall erosion, especially when non-absorbable materials are used. Therefore, the use of absorbable sutures to prevent stone formation has been advocated. Absorbable suture has been reported to lead to stone formation in a patient who underwent radical prostatectomy [2].

A hanging intravesical stone on the dome of urinary bladder is rare. It implies that the patient has undergone various procedures in the neighborhood region of the urinary bladder, for example, hysterectomy or the placement of intrauterine devices [10,11]. Theoretically children have a potential risk of intravesical stone formation in adult life if they undergo reconstructive procedures such as exstrophy repair. The incidence of intrauterine devices migrating to adjacent organs ranges between 1% and 3% [3]. Relatively speaking, the incidence of sutures serving as a nidus of stone formation is lower than that of intrauterine devices. A search of the MEDLINE database turned up only an article reporting two patients who developed bladder stones after gynecological surgeries [10]. In both cases, the investigators found that synthetic and non-absorbable suture materials were encrusted by bladder stone. The underlying mechanism would be that the suture penetrated through the dome of urinary bladder and then caused the deposition of calcium salts [12].

A patient with an intravesical stone usually presents with lower urinary tract symptoms such as pain, urgency and/or intermittency on voiding. Those patients may be asymptomatic with normal values in the urine sediment, or their symptoms may only manifest repeatedly as dysuria and pyuria for several years [10]. Abdominal radiography may reveal most intravesical stones. However, radiolucent stones would be missed by a plain X-ray film. Ultrasonography is useful for primary evaluation of intravesical stone formation [13]. Vesical calculi usually accumulate in the bottom of the urinary bladder because of gravitation. If the stone is found by ultrasonography to be constantly hanging in the dome of the urinary bladder, the implication might be that the suture material has migrated into the urinary bladder.

The sutures and vesical stones usually are removed cystoscopically without post-operative complications. For sutures that are difficult to retrieve, Bagley and his colleague have reported the successful application of neodymium-doped yttrium aluminum garnet (Nd:YAG) and holmium:YAG (Ho:YAG) lasers to divide sutures *in vitro*[14]. Conlin, a co-author of Bagley’s paper, considered that the Ho:YAG laser is safer and more effective than the other laser for intraluminal lithotripsy in the upper urinary tract [15], but the Ho:YAG laser failed to divide the GOR-TEX Suture (WL Gore & Associates, Flagstaff, AZ, USA) [14].

Intravesical stone formation induced by surgical procedures outside the urinary bladder seems to be rare. However, the complication can be prevented by taking three steps: (1) the exclusive use of absorbable sutures, such as poliglecaprone, polyglactin, polydioxanone; (2) careful avoidance of needles and sutures through the whole layer of the urinary bladder; and (3) the use of cystoscopy to check the urinary bladder.

## Conclusions

A hanging intravesical calculus on the dome of the urinary bladder is rare. It usually hints that the bladder stone encrusts sutures or devices. The complication can be prevented by the routine use of absorbable material in sutures outside the urinary bladder, no use of any suture through the urinary bladder and cystoscopic double-checking.

## Consent

Written informed consent was obtained from the patient for publication of this case report and accompanying images. A copy of the written consent is available for review by the Editor-in-Chief of this journal.

## Competing interests

The author declares no competing interests.
